# High Adherence to Iron/Folic Acid Supplementation during Pregnancy Time among Antenatal and Postnatal Care Attendant Mothers in Governmental Health Centers in Akaki Kality Sub City, Addis Ababa, Ethiopia: Hierarchical Negative Binomial Poisson Regression

**DOI:** 10.1371/journal.pone.0169415

**Published:** 2017-01-27

**Authors:** Bisratemariam Gebreamlak, Abel Fekadu Dadi, Azeb Atnafu

**Affiliations:** 1 Teklaymanot General Hospital, Addis Ababa, Ethiopia; 2 Institute of Public Health, College of Medicine and Health Sciences, University of Gondar, Gondar, Ethiopia; Helsingin Yliopisto, FINLAND

## Abstract

**Background:**

Iron deficiency during pregnancy is a risk factor for anemia, preterm delivery, and low birth weight. Iron/Folic Acid supplementation with optimal adherence can effectively prevent anemia in pregnancy. However, studies that address this area of adherence are very limited. Therefore, the current study was conducted to assess the adherence and to identify factors associated with a number of Iron/Folic Acid uptake during pregnancy time among mothers attending antenatal and postnatal care follow up in Akaki kality sub city.

**Methods:**

Institutional based cross-sectional study was conducted on a sample of 557 pregnant women attending antenatal and postnatal care service. Systematic random sampling was used to select study subjects. The mothers were interviewed and the collected data was cleaned and entered into Epi Info 3.5.1 and analyzed by R version 3.2.0. Hierarchical Negative Binomial Poisson Regression Model was fitted to identify the factors associated with a number of Iron/Folic Acid uptake. Adjusted Incidence rate ratio (IRR) with 95% confidence interval (CI) was computed to assess the strength and significance of the association.

**Result:**

More than 90% of the mothers were supplemented with at least one Iron/Folic Acid supplement from pill per week during their pregnancy time. Sixty percent of the mothers adhered (took four or more tablets per week) (95%CI, 56%—64.1%). Higher IRR of Iron/Folic Acid supplementation was observed among women: who received health education; which were privately employed; who achieved secondary education; and who believed that Iron/Folic Acid supplements increase blood, whereas mothers who reported a side effect, who were from families with relatively better monthly income, and who took the supplement when sick were more likely to adhere.

**Conclusion:**

Adherence to Iron/Folic Acid supplement during their pregnancy time among mothers attending antenatal and postnatal care was found to be high. Activities that would address the above mentioned factors were highly recommended to ensure the sustainability of mothers’ adherence to the supplement.

## Background

Anemia is a global public health problem affecting two billion people worldwide. Almost half of all preschool children, pregnant women, and close to one-third of non-pregnant women are anemic Worldwide[1]. Africa has the largest number of women with anemia next to South and Southeast Asia[1] According to the 2011 report of Ethiopian Demographic and Health Survey, moderate and mild types of anemia are abundant among the pregnant and non-pregnant mothers in the country, where, 1.2% of the cases are severe[2].

Both Folic Acid and Iron deficiency during pregnancy are risk factors for anemia, preterm delivery, low birth weight, and this contributes to the poor neonatal health and increased maternal mortality[3]. Studies indicate that mothers who receive antenatal care service will have infants with a reduced risk of neonatal deaths[4].

One of the major interventions to prevent anemia and folic acid deficiency is Iron Folic acid (IFA) supplementation. World Health Organization(WHO) recommends that all pregnant women should receive a standard dose of 30–60 mg Iron and 400 μg Folic acid during gestation as part of their ANC follow up[5]. Many countries aim for women to receive 90 or more tablets during pregnancy. However, in areas where the prevalence of anemia is high (>40%), the supplementation should continue for three months in the postpartum period. Other interventions such as food, water fortification, and anti-parasitic treatment are also suggested though their effectiveness is not clear[6].

The national guideline for control and prevention of micronutrient deficiencies stated daily iron supplementation for at least 6 months during pregnancy and 3 months postpartum have a significant importance. The National Nutrition Strategy (NNS) also stated a goal to enrich the number of women receiving Iron/Folic supplementation for more than 90 days during pregnancy and postpartum by 50%[7]. On top of that, The National Nutrition Program (NNP) stated as pregnant women should get routine Iron/Folic acid supplementation[2].

Even though, ANC is used as a major platform for Iron/Folic acid supplementation, its coverage nationally is only 43%. More than half of the pregnant women who were included in a study conducted nationally were not have at least one ANC visit. The utilization of the supplement in urban areas (27.2%) was better than the rural areas (15.2%)[2]. Iron/Folic Acid supplementation is affected by supply related factors and poor adherence to the supplement. Among women who were pregnant in the last five years, less than one percent received and took the ideal minimum number of tablets and had one time ANC visit[7,8]. Studies on the level of adherence and factors associated were limited in number[9–13] and showed discrepancy within the area and among different populations[14–16]. Therefore, this study was conducted to estimate the number of Iron/Folic Acid uptake, the level of adherence, and its associated factors among mothers during their antenatal care follow up period.

## Methods

### Study design and setting

Institutional based cross-sectional study was conducted from March up to May 2015. The study was conducted on mothers attending ANC and PNC follow up in governmental health centers in Akaki kality sub city. These mothers who were attending ANC and PNC follow up were asked about their Iron/Folic Acid uptake during their pregnancy time. The sub city is administratively subdivided into 11 woredas. The study was conducted at six governmental health centers. At the time of data collection, there were 63,311 women of childbearing age in the sub city. According to the last six months report, 1,823 pregnant women were enrolled in ANC and 2,717 mothers were enrolled in PNC services. In Ethiopia Iron/Folic Acid supplementation is the vital component of ANC and the tablet is supplemented free of charge.

### Study population and sampling procedure

The study populations were all third-trimester pregnant women and all post-natal mothers attending ANC and PNC services at all governmental health centers in Akaki Kality sub city during the data collection period. Mothers who were seriously ill at the time of data collection and mothers who were on the treatment of anemia were excluded. The necessary sample size (n) was computed by single population proportion formula [n = [(Z_a/2_)^2^*P (1-P)]/d^2^] by assuming 95% confidence level of Z α/_2_ = 1.96, margin of error 5%, design effect 2, proportion (p) of Iron/Folic Acid adherence rate 38.7%[1], and non-response rate of 5%. After considering a sample size for the second objective, which is identifying factors associated with the number of Iron/Folic acid supplement, a total sample size of 626 was considered. A systematic random sampling was used to select every second mother(K = 2) arriving at the ANC clinic in every health institutions included in the study. The sampling interval (K) was determined based on the client daily flow.

### Data collection and quality control

Data was collected using pre-tested, structured, and interviewer administered questioner that consisted of demographic data, iron supplement data, and pregnancy and healthcare system related data. Mothers who were pregnant and attend antenatal care services after third-trimester and who already gave birth and came for postnatal care services were assessed about their Iron/Folic accide supplementation practice during their pregnancy time using their recall response. Adherence is then considered as taking an Iron/Folic acid supplement for at least 90 days[5] during the first 6 months at pregnancy time. Six trained Diploma Nurses were collected the data. The trained supervisor and the principal investigator supervised the data collection process. Before the data entry, the questioner was checked for completeness.

These are some of the potential confounding variables collected and included in the analysis: Age of the mother, religion, occupation of the mother and father, educational level of the mother and the father, household income, number of pregnancy the mother had, number of children the mother had, gestational age, first ANC visit, overall ANC visit made by the mother, other illnesses the mother reported, place where the supplement was obtained, their knowledge about the supplement, distance of the health facility from their home, and weather they encountered shortage of the supplement or not.

### Data analysis

Data was checked, coded and entered to Epi-info version 3.5.1 and was exported to R version 3.2.0 for analysis. The principal investigator made data entry. Descriptive statistics such as frequency and cross tabulation were calculated for selected variables. Negative Binomial Poisson Regression Model was fitted, after testing an assumption for equal mean and variance of the corresponding count data, to model factors associated with a number of Iron/Folic Acid uptake by the mothers on the follow-up.

Hierarchical model was used: On the first step, socio-demographic variables (age, educational level, the occupation of the mothers and the fathers, income of the household, and distance to the health center) variables were fitted. The second model was fitted with maternal related conditions like a number of pregnancies, a number of children, ANC visit, whether or not the women encountered any side effect, in addition to the variables significant in the first model. The third (final) model was built with maternal awareness toward the supplement and the variables significant in the second model. In all respectively fitted models, a p-value < 0.1 was considered for selection of variables for the next model. Adjusted Incidence Rate Ratio (ARR) with its 95% confidence interval (CI) was used to report an association between number of Iron/Folic Acid uptake and identified variables. Variables having a p-value ≤ 0.05 in the final model were considered as statistically significant. Model with the best fit for the data was selected using respective model AIC.

### Ethical considerations

Ethical clearance was obtained from the institutional Ethical review Board (IRB) of the university of Gondar. Permission letter was obtained from Akaki kality health bureau and their respective offices. The purpose of the study was explained to the study participants and written consent was obtained. For those participants age less than 18 years, the written consent was obtained from their guardians. Confidentiality was maintained at all levels of the study by avoiding the use of names and other identifiers. Health education was given for the participants to increase their awareness and to improve their adherence levels.

## Results

### Socio-demographic characteristics of the respondents

Five hundred fifty-seven (557) mothers attending ANC and PNC follow-up were included in the study giving a response rate of 88.9%. The mean (±SD) age of the mother was 25.79 (±4.318) years. Most of the respondents (74.1%) were orthodox Christianity followers. Almost all of the mothers (98.8%) were married. The highest proportion of women (40.2%) had primary education. More than half of the study participants (64.6%) were homemakers, whereas 17.1% were a private employee. Most of the husbands of these women were private employees (61.9%) and the household mean (±SD) monthly income was 3,200.34 Ethiopian birr (±1544.56 birr). (Table 1)

**Table 1:** Socio-demographic characters of pregnant and postnatal mothers attending ANC and PNC follow up in governmental health centers in Akaki Kality sub city, 2015.

Variables	Number	Percentage (%)
**Age group(N = 557)**		
15–20	34	6.1
20–24	178	32
25–29	232	41.7
30–34	85	15.2
34–39	28	5
**Religion(N = 557)**		
Orthodox	415	74.5
Catholic	6	1.1
Muslim	88	15.8
Protestant	48	8.6
**Educational status of the mother(N = 557)**		
Illiterate	36	6.5
Can read and write	66	11.8
Primary education	224	40.2
Secondary education	172	30.9
College and above	59	10.6
**Occupation of the mother(N = 557)**		
House wife	360	64.6
Government employee	46	8.3
Private employee	95	17.1
Merchant	50	9
Farmer	6	1
**Husband’s education(N = 557)**		
Illiterate	16	2.9
Can read and write	26	4.7
Primary education	178	32
Secondary education	227	40.1
College and above	110	19.7
**Husband’s occupation(N = 557)**		
Government employee	118	21.2
Private employee	345	61.9
Merchant	80	14.4
Farmer	14	2.5
**Household Monthly income in ETB (N = 557)**		
900–1800	56	10.1
1801–2700	212	38.1
2701–3600	136	24.4
3601–4500	52	9.3
>4501	101	18.1

*ETB- Ethiopian birr

### Pregnancy and supplement related conditions

Out of interviewed study participants, 39% had only one child. Thirty percent (30%) of the study participants had four ANC visits. More than ninety percent of study participants (92.5%) said that taking the supplement is important. Around half of the women (59.4%) got the supplement from a pharmacy outside the health center. Of the women that were included in the study, 38.1% took the supplement for less than thirty days. More than half percent of the study participants had encountered side effects. Seventy percent (70%) of the study participants were aware of the cause, consequence, and prevention of anemia.

The majority of the study participants live nearby the health center that took them less than thirty minutes of walk. More than half of the women reported as they encountered Iron/Folic Acid shortage at the health centers. Most of the participants reported that they received health education at the health centers. (Table 2)

**Table 2:** Pregnancy and supplement related conditions of mothers attending ANC and PNC follow up in governmental health centers in Akaki Kality sub city, 2015.

Variables	Number	Percentage (%)
**No of previous Antenatal care visits made (N = 557)**		
Once	2	0.4
Twice	161	28.9
Three times	132	23.7
Four times	166	29.8
Greater than four times	96	17.2
**Made first Antenatal care visit(N = 557)**		
0–16 weeks	385	69.1
17–24 weeks	157	25
25-28weeks	30	5.2
29-36weeks	5	0.7
**Gestational age in months(N = 331)**		
6 months	81	24.5
7 months	80	24.2
8 months	98	29.6
9 months	72	21.7
**Supplement is important(N = 557)**		
Yes	515	92.5
No	42	7.5
**Supplement prevents anemia(N = 557)**		
Yes	343	61.6
No	214	38.4
**Supplement is taken for a month(N = 557)**		
Yes	180	28.7
No	397	71.3
**How were you taking the supplement(N = 557)**		
Daily	555	99.6
Every other day	2	0.4
**The supplement increases blood(N = 557)**		
Yes	285	51.2
No	272	48.5
**The supplement makes the fetus big(N = 557)**		
Yes	210	37.7
No	347	62.3
**Side effect(N = 557)**		
Yes	325	58.4
No	252	41.6
**Duration of the supplement taken(N = 557)**		
0–30 days	214	38.1
31–60 days	207	37.5
61–90+ days	136	24.4
**Adherence level(N = 557)**		
Adhered	335	60.1
Not Adhered	222	39.9
**Distance of the Health Center from home in minutes(N = 557)**		
5–15	306	54.9
16–30	209	37.5
>30	42	7.6
**Iron/Folic Acid encountered shortage at the center(N = 557)**		
Yes	331	59.4
No	226	40.6
**Received health education(N = 557)**		
Yes	397	71.3
No	160	28.7

### Adherence and factors associated with number of Iron/Folic Acid uptake by the mothers

Among 557 mothers interviewed, more than 90% were supplemented with at least one Iron/Folic Acid supplement from pill in a week during pregnancy time. Adherence, which was considered as taking of Iron/Folic Acid supplements for four or more times a week during pregnancy time, was found to be 60.1% (95%CI, 56%—64.1%).

Most of those who did not adhere stated side effect as the main reason. Heartburn was the frequent side effect reported by 96.6% of non-adhered mothers while the rest also reported vomiting (1.6%), constipation (1.3%), and diarrhea (0.5%).

Hierarchical negative binomial Poisson regression model were fitted systematically to identify factors associated with a number of Iron/Folic Acid uptake by the mothers. In the final model, the incidence of taking more number of Iron/Folic Acid supplement by the mother was higher among mother’s who had achieved secondary education(IRR_A_:1.34, 95%CI, (1.01, 1.75), who were private employee (IRR_A_:1.28, 95%CI, 1.11,1.49), who had received health education (IRR_A_: 1.17, 95%CI, (1.03, 1.32), and who thought Iron/Folic Acid supplement increases blood (IRR_A_: 1.16, 95%CI, 1.04, 1.31). On the other side, the incidence of taking more number of Iron/Folic Acid supplement by the mother was lower among mothers who reported any side effect (IRR_A_: 0.81, 95%CI, (0.73, 0.90), among mothers’ whose monthly income was in category of 135–180 USD(IRR_A_: 0.74, 95%CI, 0.60, 0.91) and 181- 225USD(IRR_A_: 0.68, 0.53, 0.88), and among mother’s who took the supplement when they were sick (IRR_A_: 0.55, 95%CI, 0.37, 0.87). (Table 3)

**Table 3:** Hierarchical model to identify factors associated with number of iron-folic acid supplement during their pregnancy period among pregnant and postnatal mothers attending ANC and PNC follow up in governmental health centers in Akaki Kality sub city, 2015.

	Model 1 ARR, 95%CI	Model 2 ARR, 95%CI	Model 3(Final) ARR,95%CI
**Mothers education(Ref = cannot read and write)**			
**Can read and write**	1.3(0.92,1.81)	1.32(0.99,1.74)	1.25(0.94,1.66)
**Primary education**	1.21(0.89,1.62)	1.17(0.91,1.48)	1.10(0.84,1.41)
**Secondary education**	1.45(1.04,1.97)*	1.42(1.09,1.82)*	1.34(1.01,1.75)*
**College and above**	1.33(0.91,1.93)	1.32(0.97,1.78)	1.22(0.88,1.67)
**Mother's occupation (Ref = Housewife)**			
**Government employ**	0.98(0.78,1.24)	0.96(0.79,1.18)	0.91(0.75,1.12)
**Private employ**	1.25(1.05,1.49)*	1.28(1.10,1.49)*	1.28(1.11,1.49)***
**Merchant**	0.97(0.77,1.22)	0.99(0.82,1.20)	0.95(0.79,1.12)
**Farmer**	0.63(0.36,1.21)	0.65(0.40,1.11)	0.63(0.39,1.07)
**Family Income(ref = 900–1800)**			
**1801–2700 ETB**	0.9(0.72,1.13)	0.82(0.67,1.00)	0.82(0.68,1.00)
**2701–3600**	0.87(0.68,1.11)	0.74(0.60,0.91)*	0.74(0.60,0.91)**
**3601–4500**	0.73(0.54,1.00)*	0.68(0.52,0.89)*	0.68(0.53,0.88)**
**>4500**	0.97(0.73,1.27)	0.82(0.65,1.03)	0.80(0.64,1.01)
**Encounter any side effect(Yes)**		0.80(0.72,0.89)*	0.81(0.73,0.90)***
**Received health education**		1.19(1.05,1.34)*	1.17(1.03,1.32)*
**The supplement increases blood(Yes)**			1.16(1.04,1.31)**
**Supplement is taken when sick(Yes)**			0.55(0.37,0.87)**

Signif. Codes:0 ‘***’ 0.001 ‘**’ 0.01 ‘*’ 0.05

(Dispersion parameter for Negative Binomial (3.0911) family taken to be 1)

Null deviance: 648.82 on 517 degrees of freedom

Residual deviance: 569.57 on 499 degrees of freedom

AIC: 4841.8

## Discussion

This study found that 99% of the subjects were prescribed the Iron/Folic Acid tablets with adherence reached to 60.9%. This result is in line with a study conducted in India in 2014[8]. However, the result is higher than the one which was reported nationally by EDHS 2011 and other national studies[2,17,18]. It is also higher than the studies done in Kenya[14] and Tanzania[3]. A possible explanation for this higher adherence might be due to most of the women (75.6%) were prescribed to take the supplement for less than sixty days. On top of that, the level of this adherence might have been overestimated as it is calculated based on the self-report of the mothers. Studies indicate that self-reporting overestimates compliance as compared to other methods of measuring adherence[18]. In contrary, the adherence might be higher since it was conducted in relatively accessible areas.

The side effect is frequently reported as a major challenge to compliance. In this Study, the side effect was the major factor that hindered adherence to the Iron/Folic Acid supplement. The incidence rate ratio of iron/Folic Acid supplementation was 19% lower among mothers who reported the side effect. Similar results were reported in studies that were done in eight rural districts of Ethiopia[7] and India[8]. In the current study, a relatively higher proportion of women with low compliance (58%) reported side effect as the reason for non-adherence. On the other hand, the incidence rate ratio of iron/Folic Acid supplement was 17% higher among mothers who received health education. This report is consistent with a study from Sweden[19], India[20], and a qualitative study from Pakistan[21]. This might happen because as knowledge of the mother related to the use of Iron/Folic Acid enhanced, they would choose to take the supplement properly.

The other variables which significantly associated with mothers’ iron/Folic Acid uptake were mother’s educational achievement and mother’s occupation. Accordingly, the incidence rate ratio of Iron/Folic Acid supplement of mothers who had completed secondary level education and employed in the private company were 34% and 28% times lower to take a number of tablets as compared to women who could not read and write and housewife, respectively. This finding is consistent with a study reported from Northern Ethiopia[18]. This might be due to education would increase the women’s access to information through reading and understanding the benefit of the supplement. Different studies reported the benefit of maternal knowledge and perception towards maternal compliance to the supplement[22–24]. Similarly, mothers with relatively better monthly income had lower incident rate ratio of taking more number of Iron/Folic Acid supplement. This finding is not consistent with the previously published literature[25–27]. This might happend due to the study design, time, population, and the way the income was measured was different among the previous and current study.

Similarly, the incidence rate ratio of taking Iron/Folic Acid supplement was 45% lower among mothers who reported they have been taking the supplement when sick. This might happen because of the fact that the number of iron/Folic Acid that should be taken according to the prescription will entirely depend on the frequency of illness that the mother had. If the mother did not have any symptom of illness, she would not take the amount of pill she should take. This could significantly reduce the number of Iron/Folic Acid supplement that should have been taken by the mother during her pregnancy period.

Lastly, if the mother perceived that the iron/Folic Acid supplement increases her blood; she had 16% more chance to take the iron/Folic Acid supplement compared to the others. This is similar with the studies reported from Senegal and Mali[25,28]. This is directly related with the content of the health education/awareness that they had from health professionals or others near to them regarding the benefit of taking the Iron/Folic Acid supplement. If they believed in the benefit of the supplement, they would take properly and this would improve their adherence and increase the number of Iron/Folic Acid they would take.

The possible limitations of this study includes that gold standard method of measuring number of Iron/Folic Acid uptake like electronic and pills counting method were not used, as they are expensive and not available. Moreover, recall bias might also be a possible explanation for this finding and should be taken into consideration while needed to use the study results. The other issue is the problem of endogeneity (missing variable bias), even though, we tried to include possible confounding variables reported from different literatures, we might not be guaranteed for an unlikely explanation of this problem.

## Conclusion and Recommendation

Adherence to Iron-folic acid supplement among women who were included in the study was found to be high as compared to other national and international studies. Getting information about Iron/Folic Acid supplement from health professionals, achieving secondary education, being private employee, and believing that the supplement increases blood were the factors that increased the number of Iron/Folic Acid uptake by the mothers. On the contrary, encountering fewer side effects, having relatively better monthly income, and taking the supplement when sick were factors that decreased the number of Iron/Folic Acid uptake by the mothers. Intervention modalities that could touch the above factors were highly recommended to sustain adherence level of the mothers to Iron/Folic Acid supplementation.
